# Medication improves velocity, reaction time, and movement time but not amplitude or error during memory‐guided reaching in Parkinson's disease

**DOI:** 10.14814/phy2.16150

**Published:** 2024-08-29

**Authors:** Michael P. Trevarrow, Miranda J. Munoz, Yessenia M. Rivera, Rishabh Arora, Quentin H. Drane, Gian D. Pal, Leonard Verhagen Metman, Lisa C. Goelz, Daniel M. Corcos, Fabian J. David

**Affiliations:** ^1^ Department of Physical Therapy and Human Movement Sciences Northwestern University Chicago Illinois USA; ^2^ Morsani College of Medicine University of South Florida Tampa Florida USA; ^3^ Division of Movement Disorders, Department of Neurology Rutgers – Robert Wood Johnson Medical School New Brunswick New Jersey USA; ^4^ Department of Neurology Northwestern University Feinberg School of Medicine Chicago Illinois USA; ^5^ Department of Kinesiology and Nutrition UIC College of Applied Health Sciences Chicago Illinois USA; ^6^ McCormick School of Engineering Northwestern University Evanston Illinois USA

**Keywords:** antiparkinson medication, motor control, Parkinson's disease, reaching

## Abstract

The motor impairments experienced by people with Parkinson's disease (PD) are exacerbated during memory‐guided movements. Despite this, the effect of antiparkinson medication on memory‐guided movements has not been elucidated. We evaluated the effect of antiparkinson medication on motor control during a memory‐guided reaching task with short and long retention delays in participants with PD and compared performance to age‐matched healthy control (HC) participants. Thirty‐two participants with PD completed the motor section of the Movement Disorder Society Unified Parkinson's Disease Rating Scale (MDS‐UPDRS III) and performed a memory‐guided reaching task with two retention delays (0.5 s and 5 s) while on and off medication. Thirteen HC participants completed the MDS‐UPDRS III and performed the memory‐guided reaching task. In the task, medication increased movement velocity, decreased movement time, and decreased reaction time toward what was seen in the HC. However, movement amplitude and reaching error were unaffected by medication. Shorter retention delays increased movement velocity and amplitude, decreased movement time, and decreased error, but increased reaction times in the participants with PD and HC. Together, these results imply that antiparkinson medication is more effective at altering the neurophysiological mechanisms controlling movement velocity and reaction time compared with other aspects of movement control.

## 1 INTRODUCTION

The loss of dopaminergic neurons in the substantia nigra pars compacta causes slowness of movement (bradykinesia) and reduced amplitude of movement (hypokinesia). Furthermore, the detriments to movement velocity and movement amplitude in people with PD are exacerbated during internally driven movements that require more cognitive control, such as memory‐guided movements (Glickstein & Stein, 1991; Jackson et al., 1995; Myall et al., 2008). Memory‐guided movements are described as internally driven because they do not rely on external feedback (Ariani et al., 2015; Debaere et al., 2003). Despite the notion that memory‐guided movements are more impaired in those with PD, substantial knowledge gaps exist in how antiparkinson medication affects memory‐guided movements.

Antiparkinson medication (e.g., levodopa) improves motor functioning and decreases the detrimental effects of PD. However, medication does not improve all aspects of movement equally. It is more effective at improving movement velocity than movement amplitude during visually guided movements (Espay et al., 2009, 2011; Velasco & Velasco, 1973). Yet, only one study has assessed the effects of medication on movement velocity and amplitude in the context of memory‐guided movements (David et al., 2022), although these movements are vital to tasks of daily living that involve goal‐oriented actions toward a remembered object (e.g., reaching for a light switch in the dark or a computer mouse while looking at the monitor). During memory‐guided reaching tasks, the participant is required to encode a target location in memory, then retain that location until a cue to execute the movement is elicited. In addition to movement amplitude and velocity, memory‐guided reaching tasks provide a viable paradigm for assessing more complex components of movement that involve cognitive control, such as reaching error. The error of the reach to the target requires several cognitive processes, including the successful encoding of the spatial location of the target into memory, the retention of that target location, and the integration of that target location into an effective motor plan (David et al., 2018, 2022).

Previously, we showed that medication did not affect movement amplitude, whereas it increased velocity and reduced error during a sequential memory‐guided reaching task (David et al., 2022). In that study, we increased the cognitive demand of the task by requiring the participant to remember 3 targets, which emphasized the spatial memory load. An alternative method to increase the cognitive demand is by increasing the temporal load, or the amount of time required to retain the remembered target location in memory (i.e., the retention delay). Increasing the retention delay can alter certain aspects of the ensuing movement. For example, increasing the retention delay reduces the accuracy of movements in people with PD and healthy controls (HC) (Ketcham et al., 2003; Trevarrow et al., 2023). However, the effect of medication on memory‐guided reaching performance with the retention delay manipulated has not been investigated. Determining the effects of retention delay, and the extent to which medication interacts with retention delay, could further elucidate the mechanisms by which these medications improve symptoms. Furthermore, reaction time and movement time are valuable measures to assess cognitive and motor processing speed, yet whether they are affected by medication during memory‐guided reaching has not been investigated. Finally, a HC group comparison can establish whether the effects of medication on memory‐guided reaching drive performance toward the performance seen in a normative population.

This investigation assessed the effects of antiparkinson medication and retention delay on memory‐guided reaching performance in a cohort of people with PD and HC participants. We hypothesized that medication would improve movement velocity and movement time during a memory‐guided reaching task but would not improve movement amplitude. Based on our previous work (David et al., 2022) and prior literature (Jahanshahi et al., 1992), we hypothesized that medication would also decrease reaching error but would not influence reaction time. Regarding retention delay, we have previously demonstrated that a shorter retention delay results in higher movement velocity and amplitude, decreased movement time, and decreased error of movement, but increased reaction time in participants with advanced PD that had subthalamic nucleus deep brain stimulation (STN‐DBS) while off medication (Trevarrow et al., 2023). Thus, we hypothesized that a short retention delay would increase movement amplitude and velocity, decrease error and movement time, and increase reaction time compared to a long retention delay.

## 2 METHODS

### 2.1 Participants

This study was conducted with approval from the Rush University Medical Center and Northwestern University Institutional Review Boards, and written informed consent was obtained from all participants. The study was done in accordance with the Helsinki Declaration of 1975. We recruited 32 participants with PD (Mean age = 65.88 ± 3.97 years, Males = 26, Females = 6) from the Rush University Medical Center Movement Disorder Clinic and Northwestern Movement Disorder Clinic. All participants were examined by a movement disorders neurologist and included in the study if they: met the UK PD Society brain bank clinical diagnostic criteria for PD (Hughes, Ben‐Shlomo, et al., 1992; Hughes, Daniel, et al., 1992), were able to understand and perform the experimental tasks, had normal or corrected visual acuity, presented no eye movement abnormalities such as double vision and/or blepharospasm, and had no other neurological comorbidities. They were also required to score ≥23 on the Montreal Cognitive Assessment (MoCA) to ensure they did not have mild cognitive impairment. Participants with PD were on at least one of the following drugs: levodopa/carbidopa, dopamine agonists, MAO‐B inhibitors, and amantadine. Each medication was converted to the Levodopa equivalent daily dose (LEDD) (Tomlinson et al., 2010).

An additional cohort of 17 HC participants was recruited. They were included in the study if they had no reported history of neurological disorders, had a score of ≤6 on the Movement Disorder Society‐sponsored revision of the Unified Parkinson Disease Rating Scale motor sub‐score (MDS‐UPDRS III) (Doyle et al., 2005), and MoCA ≥23 to exclude individuals with cognitive impairment. The cut‐off of ≤6 on the MDS‐UPDRS III for HC was determined from the mean MDS‐UPDRS III (+2 standard deviations) from HC (*n* = 196) who were part of the Parkinson's Progression Markers Initiative database (Simuni et al., 2018). Apart from these three criteria, HC met the same inclusion/exclusion criteria as those with PD. Four HC participants were excluded from our statistical analyses: one had a MoCA score of 18, one had an MDS‐UPDRS III score of 12, one was unable to attend to the instructional set and did not provide sufficient data to be included in the final analysis, and one had unusable data due to technical difficulties during the data collection. Thus, 13 HC participants were used in the final analysis (Mean age = 65.23 ± 4.34 years, Males = 11, Females = 2).

All participants were right‐handed and used their right hand to complete the memory‐guided reaching task. The Edinburgh Handedness Inventory was used to confirm right hand dominance (Oldfield, 1971). For further demographic information of the participants with PD, see Table 1.

**TABLE 1:** Participant demographics.

	Parkinson's disease	Healthy controls
Sex (M/F)	26/6	11/2
Age (mean ± SD, years)	65.88 ± 3.97	65.23 ± 4.34
MoCA (mean ± SD)	27.78 ± 1.91	27.38 ± 1.56
MDS‐UPDRS Part III, while OFF meds (mean ± SEM)	42.69 ± 2.43	3.15 ± 0.62
MDS‐UPDRS Part III, while ON Meds (mean ± SEM)	32.44 ± 2.08	–
Disease duration (years) (mean ± SD)	7.31 ± 4.36	–
LEDD (mg) (mean ± SD)	806.56 ± 675.55	–

### 2.2 Experimental conditions

For participants with PD, data collection took place over 3 days: 1 day for intake, 1 day for testing with their medication on (i.e., ON), and 1 day for testing with their medication off (i.e., OFF). During intake, participants were ON medication. They were consented, administered the MoCA (Table 1), acclimatized to the lab, and trained on the experimental tasks. Testing occurred on the next 2 days: 1 day OFF medication and 1 day ON medication, with the order of medication status randomized across participants. For OFF medication testing, participants stopped taking their medications at least 12‐hours prior to testing (Langston et al., 1992). For ON medication testing, participants took their medication as usual. To verify that participants were in the “on state”, the experimenter confirmed with the participant that they felt “on” before testing began. No participants exhibited severe dyskinesias that affected testing. For the HC, intake and experimental tasks were completed in one day.

Each session contained six different tasks examining saccades and/or reaching. The order of experimental tasks was randomized for each session. This paper focuses only on the memory‐guided reaching task. Data from four of the other five tasks has previously been published (David et al., 2022; Munoz et al., 2022, 2023, 2024).

### 2.3 Instrumentation for the memory‐guided reaching task

The memory‐guided reaching task was conducted in a completely darkened room. Participants were seated upright on an adjustable chair, with their chin on a chin rest to minimize head movement. Head and finger movements were captured with a 3‐D motion capture system (Optotrak 3020, Northern Digital, Waterloo, Ontario, Canada). Eye movements were captured at 500 Hz with a head‐mounted video‐based eye‐tracking system (Eyelink II, SR Research Ltd, Ottawa, Ontario, Canada). An active infrared emitting diode was taped to the participant's index finger to track finger movements (Northern Digital).

One target was presented to the participants using a 5‐degree of freedom robot arm (Thermo CRS, Burlington, Ontario, Canada). A central fixation light (3 mm green light‐emitting diode (LED), 70 mcd) was situated 42 cm away from the participant at 0° visual angle attached to a fixation stand. This served as the starting point for the participant's reaching motion. The robot presented the target (3 mm green LED, 70 mcd) in a plane that was 42 cm from the chin rest.

Head, finger, and robot movements were synchronized and stored using the Motion Monitor system (TheMotionMonitor, Innovative Sports Training, Chicago, IL, USA).

### 2.4 Protocol

**FIGURE 1:**
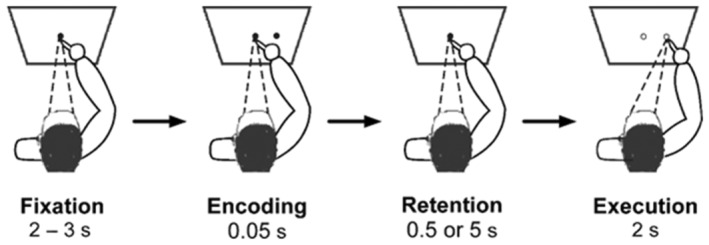
Memory‐guided reaching task. Illustration of the experimental task. The memory‐guided reaching task was performed with the right hand, and reaches were performed in 3D space. The quadrilateral depicts the plane in which the fixation and targets were presented. The unfilled circles in the execution phase depicts the extinguished fixation and target LEDs. Following the retention phase and the extinguishing of the fixation and target LEDs, participants were instructed to reach to the remembered targets as quickly as possible. Variation of the retention delay was either 0.5 or 5 s.

Each of the two testing sessions began with the administration of the MDS‐UPDRS III (Goetz et al., 2008) and was followed by the memory‐guided reaching task. The memory‐guided reaching task began with participants fixating on the central fixation light (0° visual angle) for 2–3 s with their index finger on a fixation stand. This was done to ensure that the finger and the eye focal points were at the same location in the horizontal plane. With the central fixation still lit, the robotic arm flashed one target in the participant's peripheral visual field for 0.05 s. One of two targets was presented to the right, one at 10° (linear distance of 0.074 m) or one at 15° (linear distance of 0.113 m) visual angle from central fixation. Target location was randomized. During the encoding of the target location, participants were instructed to only use peripheral vision to remember the target's location. After either a 0.5 or 5 s retention delay, the central fixation light was extinguished. This served as the cue for participants to initiate reaching to the remembered target. Participants were instructed to “reach as quickly as you can and make one smooth movement out to the remembered target.” When the reaching movement was completed, participants returned their finger to the central fixation stand, which was illuminated by a flashlight by one of the experimenters. The experimental paradigm is illustrated in Figure 1.

Participants performed one block of 40 trials per condition, in which 20 trials were performed with the short target (10°, 0.074 m) and 20 were performed with the longer target (15°, 0.113 m). Movement distance was restricted by the range over which we could record eye movements. For this study, we assessed the effects of medication and retention delay, and not the effect of target distance. Thus, we chose to only analyze trials with a longer distance, as these trials were more difficult and, thus, more sensitive to elicit differences in motor control (Fitts, 1954). Within those 20 trials, 10 had a 0.5 s delay and 10 had a 5 s delay. Prior to test trials, all participants performed sufficient practice trials until they could perform the task correctly. Practice trials were not analyzed.

### 2.5 Data analysis

The data were analyzed using a custom MATLAB script (The MathWorks 2014). A 20 Hz low‐pass 2nd order, zero‐phase Butterworth filter was applied to the finger position signals. The filtered position data was then differentiated to calculate tangential velocity.

The following procedure was used to determine the movement amplitude, movement velocity, reaching error, reaction time, and movement time. First, finger endpoints to the target were visually marked. From the visually determined finger endpoints, an algorithm searched backwards to determine the first peak in the reaching velocity profile. This peak was associated with the finger movement that brought the finger to the target location. Thus, time points corresponding to the peak were established. Next, from this peak, the algorithm searched forwards to detect the first time point when reaching velocity went below 5% of peak velocity and stayed below this threshold for 0.2 s (Kelly et al., 2002). The first time point when this condition was met was designated as the algorithmically determined finger endpoint. Next, from the first peak, the algorithm searched backwards to detect the first time point when reaching velocity went below 5% of peak velocity and stayed below this threshold for 0.2 s. The first time point when this condition was met was designated as the onset of the finger movement. The difference between this point in time and the cue to initiate the movement was defined as the reaction time, and the difference between the time corresponding to the onset of the finger movement and the time corresponding to the finger endpoint was defined as the movement time. The magnitude of the vector connecting the location of the finger at the onset of the movement and at the endpoint of the movement was defined as the movement amplitude. Once the locations of finger endpoints were determined, error was calculated by subtracting the values of these locations from the values of the corresponding target locations. Error was calculated in all three dimensions for the finger endpoints. The magnitude of finger endpoint error was calculated using the following equation:

$$\text{Finger endpoint error magnitude}=\sqrt{{\left(\text{target}\;x-\text{endpoint}\;x\right)}^{2}+{\left(\text{target}\;y-\text{endpoint}\;y\right)}^{2}+{\left(\text{target}\;z-\text{endpoint}\;z\right)}^{2}}$$

For the memory‐guided reaching task, outlier trials were removed if they had reaction times <0.2 s or >2.5 s, peak velocity >2.0 m/s, and error >0.2 m. In addition, trials where the participant looked at the target during encoding were deemed invalid and were not analyzed.

### 2.6 Statistical analysis

A paired sample *t*‐test was used to assess the effect of medication (ON vs. OFF) on the MDS‐UPDRS III. Mixed effect regression models were then utilized to assess the effect of medication and retention delay on memory‐guided reaching performance. The fixed effects were condition (medication OFF and ON), retention delay (0.5 and 5 s), and the condition by retention delay interaction. The random effect was participant. Amplitude was used as a time‐varying covariate in the velocity analysis, given the known positive relationship between movement amplitude and velocity (Corcos et al., 1989). In addition, for the finger endpoint error analysis, reaching velocity was used as a time‐varying covariate in our respective mixed effect regression models. This was done because first, a well‐known speed accuracy trade‐off (i.e., faster movements are likely to be associated with greater errors) has been established in the literature (Corcos et al., 1989; David et al., 2018; Fitts & Radford, 1966). Second, medication is very effective at improving movement velocity (Espay et al., 2011; Vaillancourt et al., 2004). Therefore, we wanted to ensure that we were assessing the effect of medication on error without the confounding effect of changes in velocity.

Separate mixed effect regression models were performed to assess the difference between the participants with PD for medication OFF and ON and HC for each outcome measure, with group (PD and HC) being the fixed effect and participant being the random effect. Finally, mixed effect regression models were used to determine the effect of retention delay in the HC participants, with retention delay (0.5 and 5 s) as the fixed effect and participant as the random effect. Normal theory methods and residual diagnostics were used to evaluate validity of assumptions. For movement velocity and reaction time, to meet the distributional assumptions for mixed modeling, the observed data were transformed using a logarithmic transformation. All statistical analyses were performed using SAS™ (version 9.4; SAS Institute, Cary, NC). All statistical tests were two‐sided, with a critical alpha of 0.05, and *p* values associated with all pairwise comparisons were corrected using the Tukey–Kramer method. The data in the text are presented as estimated means ± standard error of the mean from the mixed effect regression models. For the variables that were log‐transformed, the back transformed estimated means are reported in the text. The observed trial level data is depicted in the figures using violin plots, with the estimated means overlaid as symbols.

## 3 RESULTS

### 3.1 MDS‐UPDRS III

A paired sample *t*‐test revealed that the MDS‐UPDRS III was significantly lower in the medication ON condition in comparison with the medication OFF condition (OFF = 42.69 ± 2.43, ON = 32.44 ± 2.08, *t*_(31)_ = 4.97, *p* < 0.001), indicating that medication improved overall motor functioning.

### 3.2 Memory‐guided reaching

There were no significant interactions between medication and retention delay for any of the memory‐guided reaching outcome measures. Thus, only the main effects are reported.

### 3.3 Amplitude

**FIGURE 2:**
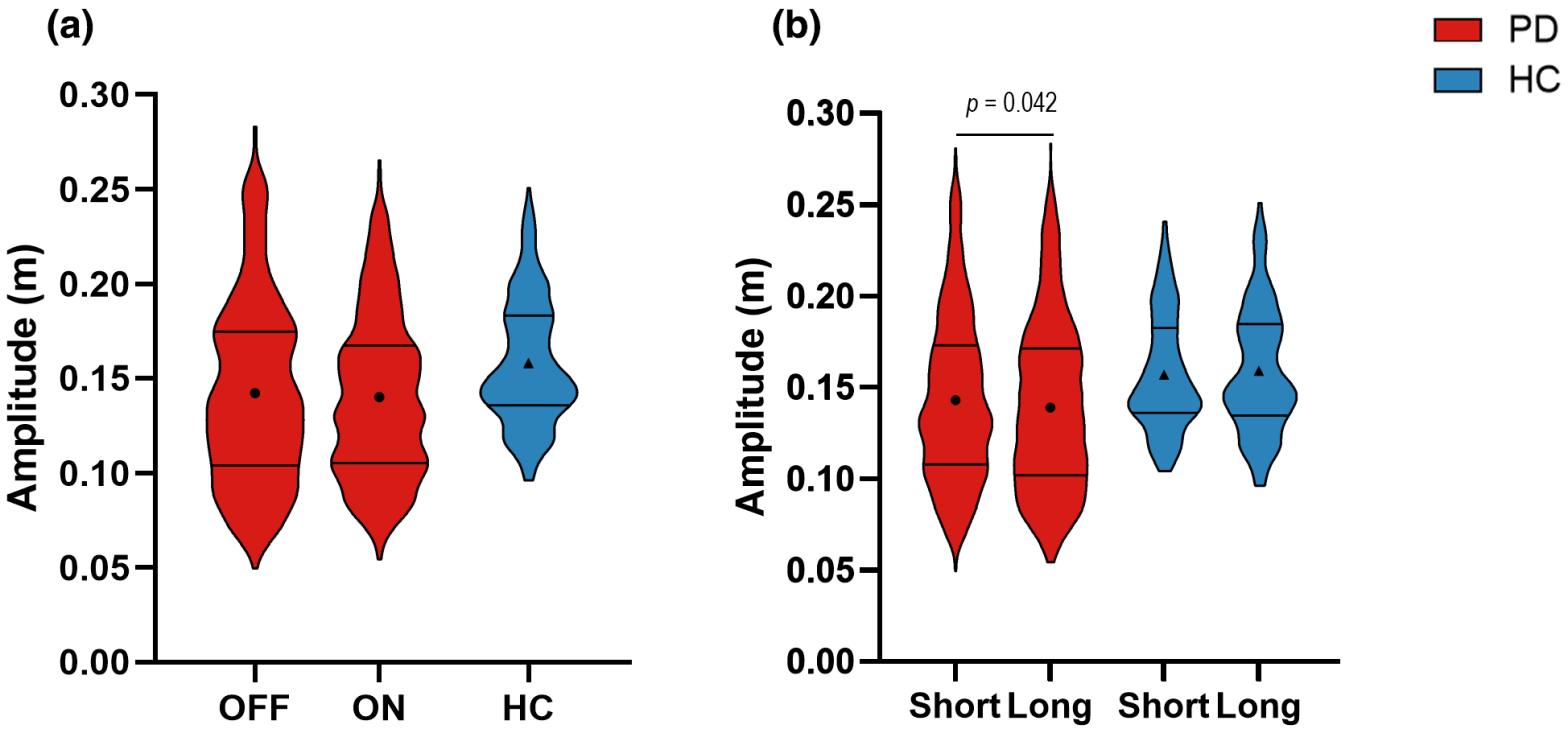
(a) Violin plots depicting the observed trial level data of amplitude for medication OFF (1st red plot), medication ON (2nd red plot), and HC (blue plot). The solid lines depict the first and third quartiles of the data, and the circles and triangles represent the estimated means for people with PD and HC, respectively. (b) The effect of retention delay on amplitude in the participants with PD (red plots) and HC (blue plots).

There was no main effect of medication on amplitude (OFF = 0.143 ± 0.007 m, ON = 0.142 ± 0.007 m, *F*_(1,1138)_ = 0.47, *p* = 0.495) (Figure 2a). There was a main effect of retention delay on amplitude (short delay = 0.144 ± 0.007 m, long delay = 0.141 ± 0.007 m, *F*_(1,1138)_ = 4.13 *p* = 0.042) (Figure 2b), indicating that the amplitude was higher during the short delay in comparison with the long delay in participants with PD.

Compared with HC, participants with PD did not have significantly different amplitude while off medication (OFF = 0.142 ± 0.007 m, HC = 0.157 ± 0.011 m, *F*_(1,770)_ = 1.22, *p* = 0.269) or on medication (ON = 0.142 ± 0.007 m, HC = 0.157 ± 0.011 m, *F*_(1,771)_ = 1.51, *p* = 0.219) (Figure 2a).

Within the HC participants, there was no effect of retention delay on amplitude (short delay = 0.156 ± 0.008 m, long delay = 0.158 ± 0.008 m, *F*_(1,215)_ = 0.23, *p* = 0.634) (Figure 2b).

### 3.4 Velocity

**FIGURE 3:**
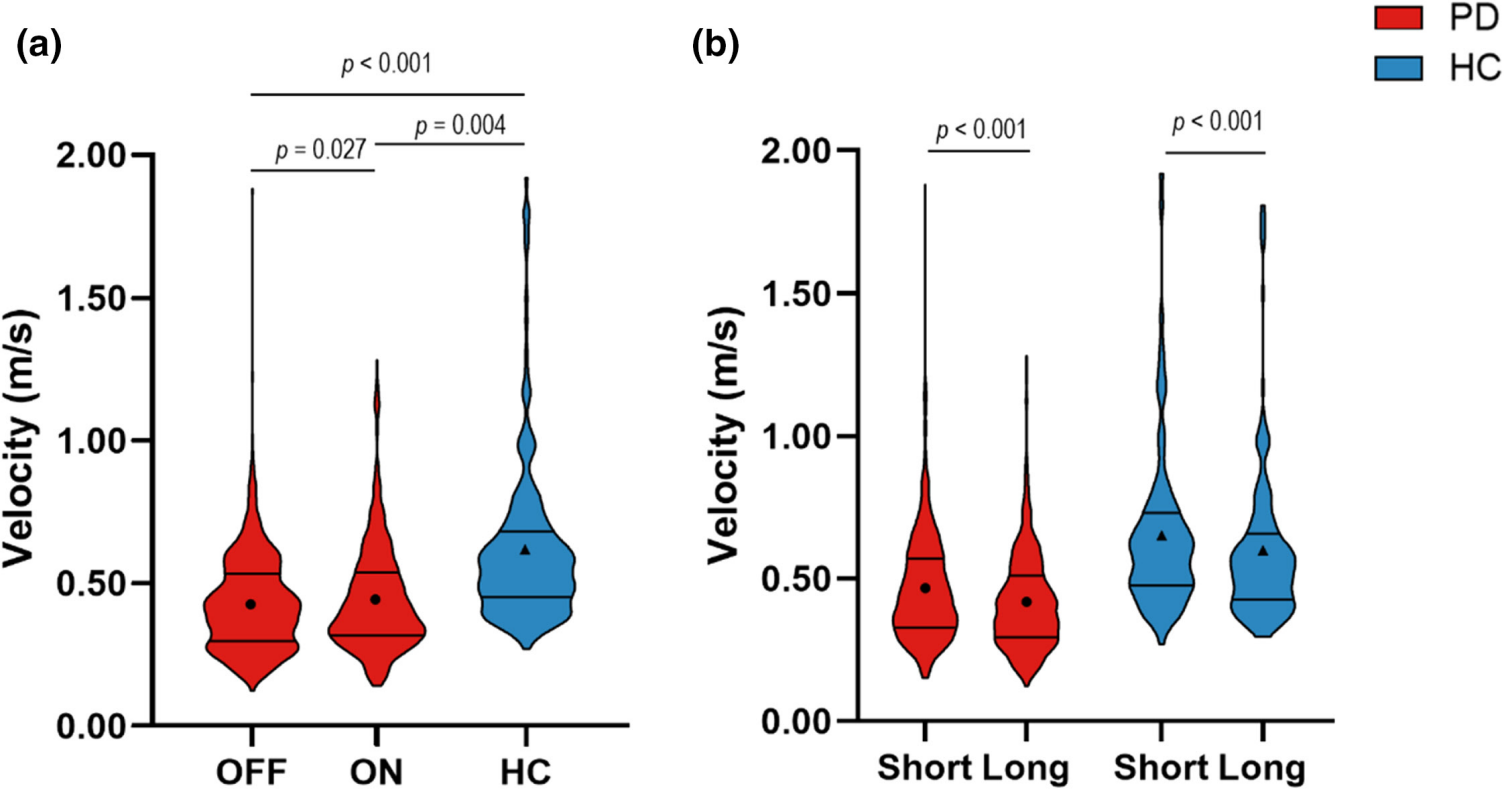
(a) Violin plots depicting the observed trial level data of velocity for medication OFF (1st red plot), medication ON (2nd red plot), and HC (blue plot). The solid lines depict the first and third quartiles of the data, and the circles and triangles represent the estimated means for people with PD and HC, respectively. (b) The effect of retention delay on velocity in the participants with PD (red plots) and HC (blue plots). Velocity was log‐transformed for statistical analysis, and the log‐transformed values are reported in the main text.

Movement velocity was logarithmically transformed to meet the distributional assumptions for mixed modeling. There was a main effect of medication on velocity (back transformed means, OFF = 0.404 m/s, ON = 0.414 m/s, *F*_(1,1137)_ = 4.93, *p* = 0.027), indicating that medication significantly increased velocity (Figure 3a). There was also a main effect of retention delay on velocity (back transformed means, short delay = 0.426 m/s, long delay = 0.392 m/s, *F*_(1,1137)_ = 55.96, *p* < 0.001), indicating that the velocity was higher during the short retention delay in comparison to the long delay (Figure 3b).

Compared with HC, participants with PD had lower velocity while off medication (back transformed means, OFF = 0.406 m/s, HC = 0.580 m/s, *F*_(1,769)_ = 11.03, *p* = 0.001) and on medication (back transformed means, ON = 0.414 m/s, HC = 0.577 m/s, *F*_(1,770)_ = 8.47, *p* = 0.004) (Figure 3a).

Within the HC participants, velocity was higher during the short retention delay compared with the long delay (back transformed means, short delay = 0.641 m/s, long delay = 0.580 m/s, *F*_(1,214)_ = 28.51, *p* < 0.001) (Figure 3b).

### 3.5 Error

**FIGURE 4:**
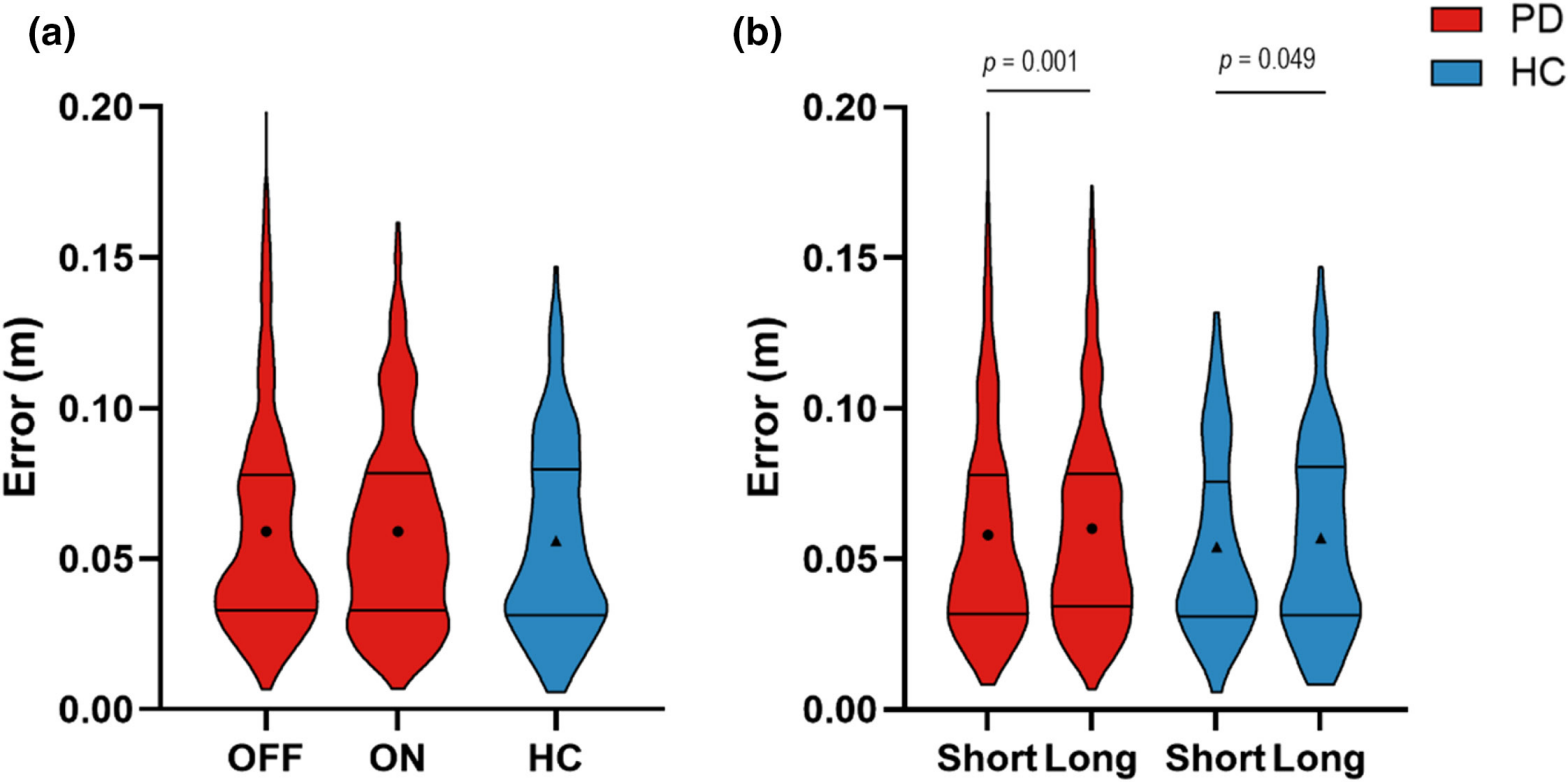
(a) Violin plots depicting the observed trial level data of error for medication OFF (1st red plot), medication ON (2nd red plot), and HC (blue plot). The solid lines depict the first and third quartiles of the data, and the circles and triangles represent the estimated means for people with PD and HC, respectively. (b) The effect of retention delay on error in the participants with PD (red bars) and HC (blue bars).

There was no main effect of medication on error (OFF = 0.060 ± 0.005 m, ON = 0.060 ± 0.005 m, *F*_(1,1137)_ < 0.01, *p* = 0.950) (Figure 4a). There was a main effect of retention delay on error (short delay = 0.058 ± 0.005 m, long delay = 0.062 ± 0.005 m, *F*_(1,1137)_ = 11.31, *p* = 0.001), indicating that error was lower during the short retention delay in comparison to the long delay (Figure 4b).

Compared with HC, participants with PD did not have significantly different error while off medication (OFF = 0.061 ± 0.005 m, HC = 0.050 ± 0.008 m, *F*_(1,769)_ = 1.14, *p* = 0.285) or on medication (ON = 0.062 ± 0.005 m, HC = 0.049 ± 0.008 m, *F*_(1,770)_ = 2.02, *p* = 0.156) (Figure 4a).

Within the HC participants, the error was lower within the short retention delay compared with the long delay (short delay = 0.051 ± 0.009 m, long delay = 0.055 ± 0.009 m, *F*_(1,214)_ = 3.91, *p* = 0.049) (Figure 4b).

### 3.6 Reaction time

**FIGURE 5:**
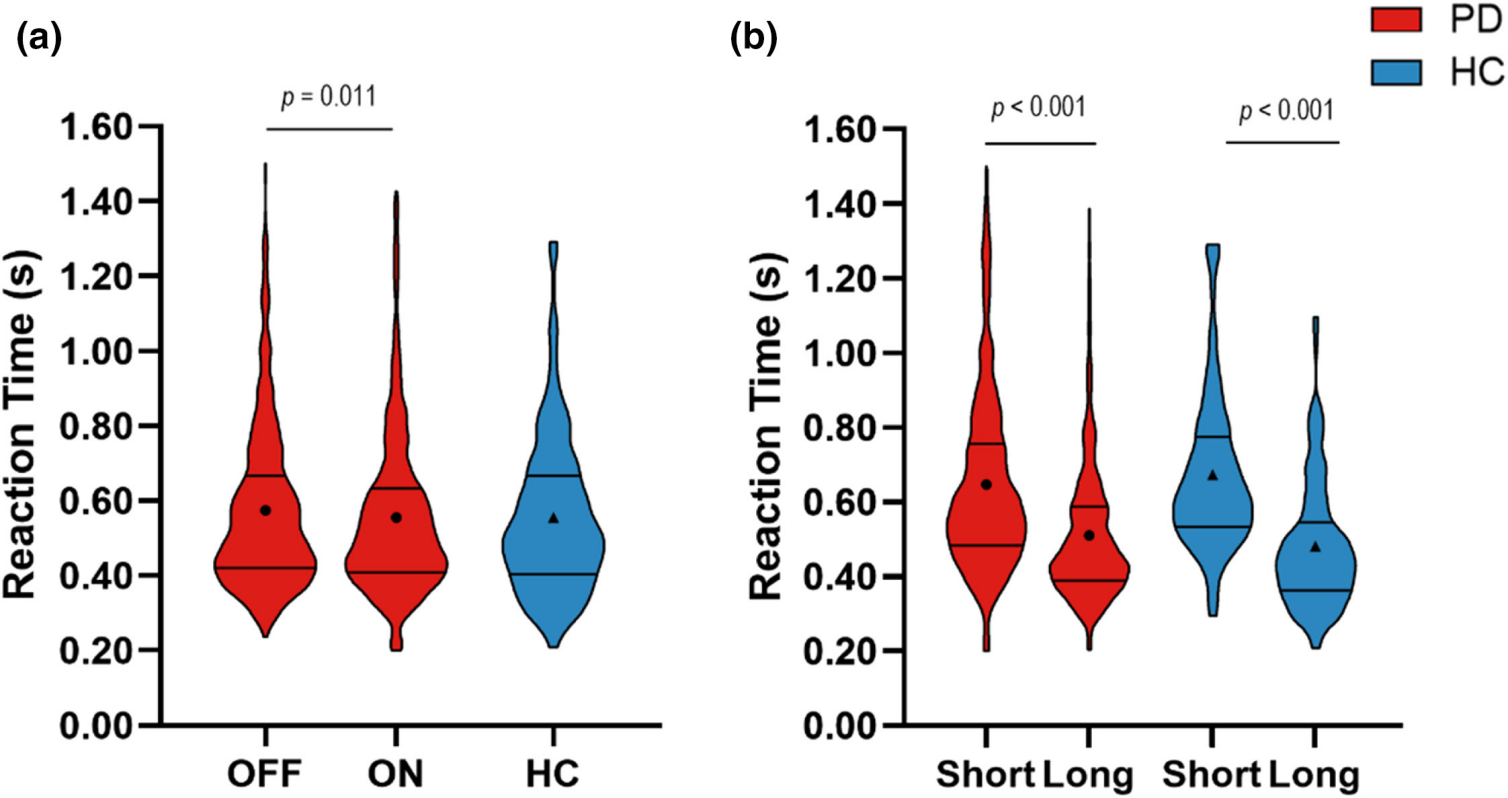
(a) Violin plots depicting the observed trial level data of reaction time for medication OFF (1st red plot), medication ON (2nd red plot), and HC (blue plot). The solid lines depict the first and third quartiles of the data, and the circles and triangles represent the estimated means for people with PD and HC, respectively. (b) The effect of retention delay on reaction time in the participants with PD (red bars) and HC (blue bars). Reaction time was log‐transformed for statistical analysis, and the log‐transformed values are reported in the main text.

Reaction time was logarithmically transformed to meet the distributional assumptions for mixed modeling. There was a main effect of medication on reaction time (back transformed means, OFF = 0.556 s, ON = 0.534 s, *F*_(1,1138)_ = 6.46, *p* = 0.011), indicating that medication reduced reaction time (Figure 5a). There was also a main effect of retention delay on reaction time (back transformed means, short delay = 0.616 s, long delay = 0.483 s, *F*_(1,1138)_ = 244.78, *p* < 0.001), indicating that the reaction time was longer during the short retention delay compared with the long delay (Figure 5b).

Compared with HC, participants with PD did not have significantly different reaction times while off medication (back transformed means, OFF = 0.543 s, HC = 0.517 s, *F*_(1,770)_ = 0.45, *p* = 0.504) or on medication (back transformed means, ON = 0.509 s, HC = 0.517 s, *F*_(1,771)_ = 0.03, *p* = 0.855) (Figure 5a).

Within the HC participants, the reaction time was longer during the short delay compared with the long delay (back transformed means, short delay = 0.643 s, long delay = 0.451 s, *F*_(1,215)_ = 108.98, *p* < 0.001) (Figure 5b).

### 3.7 Movement time

**FIGURE 6:**
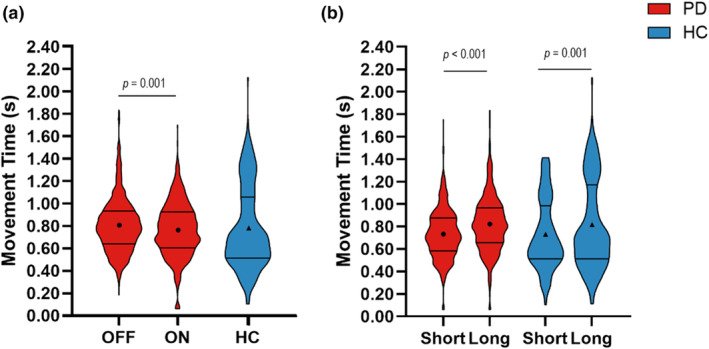
(a) Violin plots depicting the observed trial level data of movement time for medication OFF (1st red plot), medication ON (2nd red plot), and HC (blue plot). The solid lines depict the first and third quartiles of the data, and the circles and triangles represent the estimated means for people with PD and HC, respectively. (b) The effect of retention delay on movement time in the participants with PD (red bars) and HC (blue bars).

There was a main effect of medication on movement time (OFF = 0.798 ± 0.030 s, ON = 0.762 ± 0.030 s, *F*_(1,1138)_ = 11.87, *p* = 0.001), indicating that medication decreased movement time (Figure 6a). There was also a main effect of retention delay on movement time (short delay = 0.740 ± 0.030 s, long delay = 0.820 ± 0.030 s, *F*_(1,1138)_ = 58.71, *p* < 0.001), indicating that the short retention delay decreased movement time compared with the long delay (Figure 6b).

Compared with HC, participants with PD did not have significantly different movement times while off medication (OFF = 0.810 ± 0.042 s, HC = 0.783 ± 0.066 s, *F*_(1,770)_ = 0.12, *p* = 0.730) or on medication (ON = 0.746 ± 0.044 s, HC = 0.783 ± 0.069 s, *F*_(1,771)_ = 0.20, *p* = 0.655) (Figure 6a).

Within the HC, the movement time was shorter during the short delay in comparison with the long delay (short delay = 0.728 ± 0.092 s, long delay = 0.817 ± 0.092 s, *F*_(1,215)_ = 12.47, *p* = 0.001) (Figure 6b).

## 4 DISCUSSION

Our results demonstrated that antiparkinson medication improved movement velocity, movement time, and reaction time, but had no effect on movement amplitude and reaching error. We also found that a shorter retention delay resulted in increased movement amplitude and velocity, decreased movement time and error, but increased reaction time. The implications of these results are discussed in detail below.

### 4.1 Medication improves movement velocity and movement time but has no effect on movement amplitude

Our results indicated that medication significantly increased movement velocity and decreased movement time toward the direction of the HC participants, while movement amplitude was unaffected. These findings corroborate several studies that have identified that medication selectively improves movement velocity (Espay et al., 2009, 2011; Velasco & Velasco, 1973), indicating that antiparkinson medication may impact a pathophysiological process within the brain/basal ganglia that is specific to the modulation of movement velocity, which in turn decreases the movement time. Beta band activity has consistently been demonstrated to be increased in strength within the subthalamic nucleus (STN), and antiparkinson medication can reduce this pathologically increased beta band activity (Cassidy et al., 2002; Doyle et al., 2005; Levy et al., 2002; Priori et al., 2004; Weinberger et al., 2006). Similarly, increased cortical beta band strength in older adults results in an increased movement time (Heinrichs‐Graham & Wilson, 2016), suggesting that a decrease in beta band strength may contribute to the velocity at which the movement is performed. Moreover, beta band activity occurs in “bursts”, and the dynamics of these beta bursts are critical for proper motor functioning (Karekal et al., 2022). Beta band bursts are increased in strength and duration in people with PD, and these alterations contribute to decreased movement velocity (Kehnemouyi et al., 2021; Lofredi et al., 2019; Torrecillos et al., 2018) but are normalized by medication (Sure et al., 2021). Together, these findings suggest that modulation of beta band power and beta band bursts by antiparkinson medication may result in selective increases to movement velocity, which in turn decreases the movement time.

The neural control of movement amplitude has been less extensively studied in comparison with other movement parameters, although several different brain regions and processes are thought to contribute to its coding. One study in humans substantiated the role of the premotor cortex in coding movement amplitude by using transcranial magnetic stimulation to demonstrate that reaching amplitude is altered when the activity within the premotor cortex is disrupted during the motor planning stage (Davare et al., 2015). Other studies have demonstrated that the basal ganglia may also play a role in coding movement amplitude (Desmurget et al., 2003, 2004). Finally, the strength of the gamma event‐related synchronization in the primary motor cortex during movement execution scales with the amplitude of the movement (Muthukumaraswamy, 2010; Tatti et al., 2023). Thus, the neural coding of movement amplitude appears to be attributed to several different mechanisms and brain regions and may not solely rely on dopaminergic processes affected by medication.

Although movement amplitude was not statistically different between the HC and people with PD, it was much larger in the HC. Indeed, hypokinesia is a common feature of PD. Several contributing factors could result in reduced movement amplitude during memory‐guided reaching. First, a perceptual contraction of the endpoint positions may contribute to altered amplitude and error (McIntyre et al., 1998). Possibly, people with PD may experience a general contraction of their perceptual space, limiting the ability to effectively encode the location of the target. Second, people with PD have notable disruption in their ability to encode and integrate proprioceptive feedback (Adamovich et al., 2001; David et al., 2022). Disrupted proprioceptive feedback could contribute to a disruption of the internal model, in which sensory feedback from the actual movement is not being accurately compared with the desired outcome of the movement, resulting in inaccurate movements.

### 4.2 Medication has no effect on reaching error

Our results indicated that medication did not significantly improve reaching error toward the direction of HC. Performing an accurate reach requires successful integration of several cognitive processes, including correctly encoding the target location, retaining it in memory, and integrating that target location into an accurate motor plan. In memory‐guided tasks, maintenance of a target location during the retention delay is associated with persistent activity within the prefrontal cortex (Curtis et al., 2004; Riley & Constantinidis, 2015). Additionally, the posterior parietal cortex is implicated in maintaining spatial representations and integrating visuomotor transformations (Batista et al., 1999; Buneo et al., 2002; Merriam et al., 2003; Sereno et al., 2001). Previous literature has shown that people with PD have reduced activity in the ventrolateral prefrontal cortex during internally driven movements in comparison to externally driven movements, whereas HC showed the opposite pattern of activity (Martinu et al., 2012). Medication did not alter this pattern of activity in the people with PD, implying that at least some cortical regions that are implicated in the control of internally driven movements are not robustly affected by antiparkinson medication. However, previous literature has also shown that antiparkinson medication can normalize regional blood flow within the dorsolateral prefrontal cortex during a spatial working memory task (Cools et al., 2002). Thus, future work should continue to assess the effects of antiparkinson medication on brain regions implicated in internally driven movements in order to better elucidate their effects on memory‐guided reaching performance.

Alternatively, the reaching error may have been similar in the medication ON and OFF conditions because our instructions to the participants emphasized reaching quickly but not accurately. We previously demonstrated that medication significantly reduced reaching error and increased movement velocity during a sequential memory‐guided reaching task in which we emphasized performing the reach accurately (David et al., 2022). Thus, the effect of medication on velocity may be robust enough that it is present regardless of task instructions. On the other hand, performing the task with the instruction to reach as accurately as possible may be necessary to uncover a difference in the amount of reaching error between the medication ON and medication OFF conditions.

### 4.3 Medication decreases reaction time

Our results indicated that medication significantly decreased reaction time in people with PD toward the direction of HC. Previous research has demonstrated that antiparkinson medication in people with PD had no effect on reaction times during a variety of simple and choice reaction time tasks (Jahanshahi et al., 1992), suggesting that the effect of medication on reaction time may be specific to memory‐guided movements. Memory‐guided movements are internally driven and largely controlled by regions within the basal ganglia (Cunnington et al., 2002; Filyushkina et al., 2019). While off their medication, people with PD exhibit increased activation within the putamen, ventral thalamus, and subthalamic areas during internally driven movements compared with HC (Filyushkina et al., 2019). Potentially, antiparkinson medication helps to restore normal activity within areas of the basal ganglia that contribute to internally driven movements, which in turn may result in enhanced movement facilitation and decreased reaction time. Similarly, a decrease in beta band power is necessary for movement disinhibition (Heinrichs‐Graham & Wilson, 2015, 2016). Thus, the medication induced reduction in beta band activity and associated reduction in movement inhibition may result in a shortened reaction time.

### 4.4 Healthy control comparisons

While people with PD had significantly lower velocity compared with HC, the rest of the motor measures were not significantly different between the groups. However, when looking at the data in the figures, at least some of the measures appear to be quite different between the two groups. For example, movement amplitude was much greater in the HC (~12%) compared with the people with PD. The lack of statistically significant differences between the two groups could be a result of the decreased statistical power in the between‐subjects comparisons versus the within‐subjects comparisons. The between‐subjects comparisons have reduced power due to the increased variability between the groups (as opposed to the variability across conditions in the within‐subjects comparison).

Additionally, we studied memory‐guided reaching at 11.3 cm amplitude. Potentially, longer movement distances might have elicited greater deficits in memory‐guided reaching in people with PD. For example, one study demonstrated that people with PD have increased difficulty in maintaining a desired force output when external visual feedback is removed (Vaillancourt et al., 2001). This deficiency becomes more pronounced with greater levels of force that need to be maintained. Thus, in the current study, longer movement distances would likely increase velocity and require greater muscle force, revealing greater deficits in movement velocity, amplitude, error, and reaction time. These longer reaches may then be better suited to reveal improvements in movement velocity, amplitude, and error with dopaminergic medication. Thus, future work should focus on determining whether people with PD show increased deficits with greater reaching distances, and whether medication proves beneficial under these circumstances.

### 4.5 A shorter retention delay results in increased movement velocity and amplitude, decreased error and movement time, but increased reaction time

Our results indicated that a shorter retention delay increased movement velocity and reduced movement time. This is in line with results from our previous study (Trevarrow et al., 2023) and expands on them by demonstrating that this phenomenon occurs regardless of medication status. Similar effects of retention delay were seen in the HC group, implying that the way in which these aspects of movement are impacted by the retention delay are likely a result of physiological mechanisms that remain intact in participants with PD. Essentially, a longer retention delay can result in degradation of the memorized target location, resulting in uncertainty about the target's location and movement plan, which may contribute to a slower reach with more error. Additionally, the large increase in reaction time during the short retention delay is a physiological phenomenon that has been previously reported (Churchland & Shenoy, 2007). This may have occurred because the shorter delay was too brief to encode the target location and form the sensorimotor plan before the cue to move was elicited. Longer retention delays, on the other hand, are thought to provide more time prior to the cue to plan the movement, saving time later and resulting in a shorter reaction time (Churchland & Shenoy, 2007; Crammond & Kalaska, 2000; Riehle et al., 1994; Rosenbaum, 1980).

## 5 CONCLUSION

We utilized a memory‐guided reaching task to demonstrate that antiparkinson medication increases movement velocity, decreases movement time, and decreases reaction time, but has no effect on movement amplitude or error. This is the first study to demonstrate these results in an internally driven, memory‐guided reaching task with the retention delay manipulated. We also demonstrated that a shorter retention delay results in increased movement velocity and amplitude, decreased movement time, and reduced error, but increased reaction time. Overall, these findings suggest that antiparkinson medication may act on distinct neurophysiological mechanisms, leading to differential effects on various components of cognitive‐motor control. Understanding the distinct neurophysiological mechanisms that result in differential effects of antiparkinson medication on various aspects of motor control is critical to gain insight into the development of more specialized therapeutic interventions that serve to improve the array of motor and cognitive symptoms in people with PD.

## FUNDING INFORMATION

This study was supported by National Institutes of Health (R56 NS040902, R01 NS09295001, F31 NS12069501, T32 NS047987, and T32 HD07418).

## CONFLICT OF INTEREST STATEMENT

Authors FD, MM, and MT received grant support from the NIH. Author GP received grant support from the NIH and consults for Guidepoint and Kyowa Kirin. Author LV M receives honoraria for consulting services/advisory boards from AbbVie, Abbott, and Supernus, and research support from Abbott, Neuroderm, and NIH. Author DC has stock ownership in Medtronic, consultancies with University of Illinois Chicago and Council for Jewish Elderly SeniorLife, honoraria from MDS, 2021 Shanghai International Symposium on Medicine in the 21st Century, ACSM, Marianjoy Rehabilitation Hospital, and MDS‐PAS 2022, received grant support from the NIH and Michael J. Fox foundation, and receives lecture and reviewer fees from the NIH. No other authors have conflicts of interest to report.

## ETHICS APPROVAL AND CONSENT TO PARTICIPATE

The Institutional Review Board at Northwestern University and Rush University Medical Center approved this study (IRB # STU00202007). Written informed consent was acquired from all participants. This study was done in accord with the Helsinki Declaration of 1975.

## Data Availability

The data supporting the findings of this study are available upon reasonable request to the corresponding author.
